# Supplementary material for: Using the Aqueous Phase Produced from Hydrothermal Carbonization Process of Brown Seaweed to Improve the Growth of Phaseolus vulgaris
Source: Plants (Basel). 2023 Jul 24;12(14):2745. doi: 10.3390/plants12142745 (PMC10383230; doi:10.3390/plants12142745)
Supplement: Supplementary file 1 [file plants-12-02745-s001.zip › Figure S1.pdf]

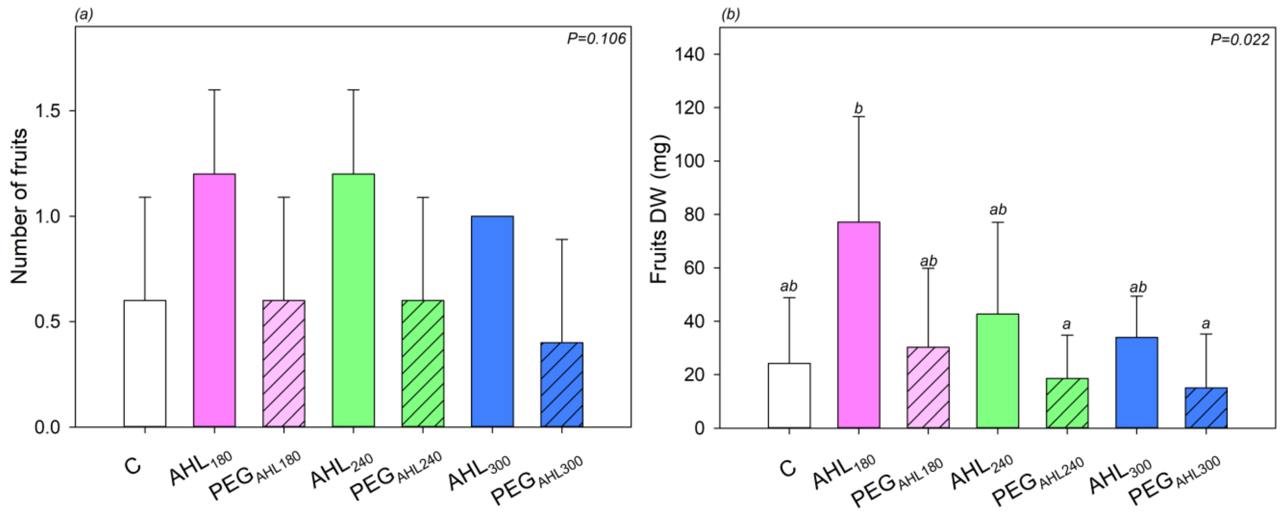

Figure S1: Mean value ± SD (n=5) of (**a**) number of fruits and (**b**) fruits dry weight (Fruits DW) recorded in samples which seed were soaked into water (control samples, C), AHL_{180} solution and the corresponding PEG isotonic solution (AHL_{180} and PEG_{AHL180} samples, respectively), AHL_{240} solution and corresponding PEG isotonic solution (AHL_{240} and PEG_{AHL240} samples, respectively), AHL_{300} solution and corresponding PEG isotonic solution (AHL_{300} and PEG_{AHL300} samples, respectively). Different letters indicate statistically different values based on One-way Anova test. *P* values are reported.
